# HEALTH PRIORITIES ALIGNED DECISION MAKING FOR DISCRETE DECISIONS

**DOI:** 10.1093/geroni/igad104.2965

**Published:** 2023-12-21

**Authors:** Jack Banks, Suha Soni, Vanessa Madrid, Rafael Samper-Ternent, Salman Arain, Mary Tinetti, Aanand Naik

**Affiliations:** University of Texas Health Science Center at Houston, Houston, Texas, United States; University of Texas Health Science Center at Houston, Houston, Texas, United States; University of Texas Health Science Center at Houston, Houston, Texas, United States; UTHealth Houston, Houston, Texas, United States; University of Texas Health Science Center at Houston, Houston, Texas, United States; Yale School of Medicine, New Haven, Connecticut, United States; University of Texas Health Science Center at Houston, Houston, Texas, United States

## Abstract

The outcomes that establish the evidence-base and frame expectations for discrete interventions, primarily survival and disease-specific markers, may not be the outcomes older adults with multiple chronic conditions (MCCs) prioritize. An advisor group consisting of 6 health professionals with expertise in geriatrics, cardiology, vascular surgery and decision-making plus a panel of patient stakeholders created a decision-making framework titled ‘health priorities identification – discrete intervention decision’ (HPI-DID) process. This study aimed to refine the HPI-DID process using revascularization for peripheral arterial disease (PAD) as an exemplar procedure through eliciting feedback on the HPI-DID process from patients and caregivers. Seven older adults who recently underwent a revascularization procedure for PAD or their caregivers took part in one-to-one interviews with JB and SS. During interviews, participants were asked to verbalize or ‘think-aloud’ their thoughts while reviewing and performing each component of the health priorities process and probed what could be added or cut to improve the procedure-related communication and decision-making process. Participant feedback resulted in structural changes to the process. Font size was increased and specific words were bolded. Three additional tables detailing expected benefits, tradeoffs and complications from the procedure and their expected durations were added. The mean percentage of patients who experience these outcomes is now also listed. We will conduct a pilot trial to evaluate the feasibility and acceptability of our refined HPI-DID process with clinical interventionalists and older patients with MCCs considering revascularization for PAD.

